# Mild manifestations of COVID-19 in healthcare workers

**DOI:** 10.1371/journal.pntd.0008950

**Published:** 2020-12-22

**Authors:** Jingwen Li, Xi Long, Qing Zhang, Xi Fang, Na Li, Zhicheng Lin, Jinghong Li, Nian Xiong

**Affiliations:** 1 Department of Neurology, Union Hospital, Tongji Medical College, Huazhong University of Science and Technology, Wuhan, Hubei, China; 2 Department of Radiology, Union Hospital, Tongji Medical College, Huazhong University of Science and Technology, Wuhan, Hubei, China; 3 Wuhan Red Cross Hospital, Wuhan, Hubei, China; 4 Laboratory of Psychiatric Neurogenomics, McLean Hospital, Harvard Medical School, Belmont, Massachusetts, United States of America; 5 Department of Medicine, University of California San Diego, La Jolla, California, United States of America; Medizinische Universitat Wien, AUSTRIA

## Abstract

Medical staff treating Coronavirus Disease 2019 (COVID-19) patients are at high risk for exposure to Severe Acute Respiratory Syndrome Coronavirus 2 (SARS-CoV-2), and many have been infected, which may cause panic among medical workers, their relatives, health professionals, and government leaders. We report the epidemiologic and clinical characteristics of healthcare workers and that the majority of infected medical staff had milder symptoms/conditions with a better prognosis than admitted patients. Timely improvement to medical staff’s working conditions such as allowing adequate rest and providing sufficient medical protection is extremely important.

## Introduction

From the beginning of the Coronavirus Disease 2019 (COVID-19) pandemic in Wuhan, China, there were reports of Severe Acute Respiratory Syndrome Coronavirus 2 (SARS-CoV-2) infections in healthcare workers (HCWs) including doctors, nurses, and other support staff. Considering previous knowledge about Severe Acute Respiratory Syndrome (SARS), the Wuhan Red Cross Hospital (WRCH) quickly increased their stock of personal protective equipment (PPE) [1,2]. A total of 3,387 HCWs (4.8% of all HCWs involved and fatality rate of 0.6%) were reportedly diagnosed with COVID-19 by February 24, 2020 in China [3]. In the United States, the Centers for Disease Control and Prevention reported 191,649 confirmed HCWs cases of COVID-19 and 770 deaths by October 2020 [4]. HCWs in many countries are experiencing both physical exhaustion and psychological challenges [5]. Albeit at the highest risk, medical workers have always been brave, capable, and responsible in the medical and health undertakings. The clinical characteristics and outcomes of COVID-19 in HCWs have not been specifically investigated and compared with non-HCWs. We hope to increase the global vigilance and prevent more medical workers from getting infected with the virus. To address this issue, we have summarized a comparison of COVID-19 cases in HCW and non-HCW patients admitted to our hospital during the same time period.

## Milder manifestations of healthcare workers

The WRCH is located in downtown Wuhan, 1.4 km from the Wuhan Huanan Seafood Market. As 1 of the first hospitals designated to treat COVID-19 patients in January 2020, the WRCH policy was to admit only COVID-19 patients as a means to contain SARS-CoV-2 in Wuhan, China. In the following months, a total of 56 HCWs from WRCH were hospitalized for COVID-19, of which 46% were nurses, 38% were physicians, and 16% were supporting staff. We also randomly (by using a random number generator) selected 121 non-HCW patients from 728 admitted non-intensive care unit (ICU) patients from January 23 to February 20 as a nonmedical staff control. We compared the clinical characteristics and outcomes of the 2 groups, as a retrospective case series study. All patients were diagnosed with COVID-19 according to the guideline of SARS-CoV-2 (Trial Version 6 of the Chinese Government) [6]. Throat swab samples from the upper respiratory tracts of all patients were collected for extracting RNA from patients suspected of having the infection. The presence of SARS-CoV-2 was confirmed by real-time reverse transcription polymerase chain reaction (RT-PCR) assay, which was carried out in 2 different institutions: Union Hospital, Tongji Medical University, Huazhong University of Science and Technology and the clinical laboratories of ADICON Medical Laboratory Center. We used Statistical Package for the Social Sciences (IBM SPSS Statistics for Windows, version 25.0, IBM, Armonk, New York, United States of America) for all data analyses, denoting *P* values of <0.05 as statistically significant.

Compared with non-HCW patients, the HCW group had less underlying comorbidities, especially cardiovascular diseases, and fewer severe symptoms such as shortness of breath, dizziness, or high fever, which may be partly attributed to the younger age. Laboratory studies also showed less severe abnormalities, including blood lymphocyte count, prothrombin time, activated partial thromboplastin time and lactate dehydrogenase, C-reactive protein, and erythrocyte sedimentation rate. An interesting finding was the lower severity and extent of lung lesions in the medical staff than the admitted patients. Specifically, the HCW group had fewer multiple ground-glass opacity (45% versus 93%), less bilateral pneumonia (29% versus 88%), less pleural effusion (0% versus 30%), compared with the non-HCW group (*P* < 0.01) (Table 1). Treatment measures between the 2 groups were the same, including symptomatic therapy, antiviral therapy, corticosteroid therapy, and respiratory support. Due to inevitable exposure to COVID-19, our HCWs were tested for SARS-CoV-2 sooner and hospitalized earlier than non-HCWs; however, the mortality rates did not differ, 2% in both HCW and non-HCW groups (Table 1). After the beginning of March 2020, there were no new HCW COVID-19 patients in WRCH.

**Table 1:** Baseline characteristics, laboratory findings, chest CT findings, and clinical outcomes of 177 patients with COVID-19 (56 HCWs and 121 non-HCW admitted patients) at WRCH.

No. (%)	Medical staff (*n* = 56)	Hospitalized patients (*n* = 121)	*P* value
**Age (years)**			
≤29	12 (21%)	6 (5%)	**0.01**
30–39	19 (34%)	14 (12%)	
40–49	17 (30%)	24 (20%)	
50–59	8 (14%)	29 (24%)	
≥60	0 (0%)	48 (40%)	
**Sex**			
Female	36 (64%)	65 (54%)	0.19
Male	20 (36%)	56 (46%)	
**Chronic medical illness**			
Cardiovascular disease	3 (5%)	40 (33%)	**0.01**
Digestive system disease	3 (5%)	14 (12%)	0.19
Endocrine system disease	4 (7%)	15 (12%)	0.29
Malignant tumor	0 (0%)	2 (2%)	0.33
Nervous system disease	0 (0%)	2 (2%)	0.33
Respiratory system disease	0 (0%)	6 (5%)	0.09
Urinary system diseases	1 (2%)	3 (2%)	0.77
**Signs and symptoms at admission**			
Fever	24 (43%)	113 (93%)	**0.01**
Cough	35 (63%)	89 (74%)	0.14
Shortness of breath	9 (16%)	49 (40%)	**0.01**
Fatigue	21 (38%)	57 (47%)	0.23
Myalgia	11 (20%)	23 (19%)	0.92
Headache	3 (5%)	9 (7%)	0.61
Sore throat	9 (16%)	10 (8%)	0.12
Chest pain	8 (14%)	26 (21%)	0.26
Dizziness	4 (7%)	23 (19%)	**0.04**
**Chest X-ray and CT findings**			
Unilateral pneumonia	32 (57%)	9 (7%)	**0.01**
Bilateral pneumonia	16 (29%)	107 (88%)	**0.01**
Multiple motting and ground-glass opacity	25 (45%)	113 (93%)	**0.01**
Bronchitis	11 (20%)	67 (55%)	**0.01**
Pleural effusion	0 (0%)	36 (30%)	**0.01**
**Laboratory results (median, range)**			
Neutrophil percentage, %	58.17 (38.6–86.5)	66 (3.13–94.7)	**0.01**
Lymphocyte percentage, %	32.6 (8.6–50.2)	25.1 (2–54)	**0.01**
Hemoglobin, g/L	136.5 (74–173)	134 (80–314)	0.19
Lactate dehydrogenase, U/L	142.2 (15.1–337.4)	200 (103–922.2)	**0.01**
Prothrombin time, s	11.7 (1.04–17.2)	12.5 (10.6–28.2)	**0.01**
Activated partial thromboplastin time, s	25.45 (1.03–38.1)	29.9 (11.7–54.2)	**0.01**
D-dimer, mg/L	0.225 (0.1–0.86)	0.53 (0.1–48.4)	0.07
Alanine aminotransferase, U/L	15.55 (3.9–61.3)	24.1 (3.7–163.8)	**0.01**
Aspartate aminotransferase, U/L	19.95 (6.3–65.1)	29.7 (6.3–140.7)	**0.01**
C-reactive protein, mg/L	2.975 (0.13–82.82)	17.4 (0.1–310.01)	**0.01**
Erythrocyte sedimentation rate, mm/h	7 (1–54)	37 (4–76)	**0.01**
**Clinical outcome**			
Remained in hospital	18 (32%)	31 (26%)	0.66
Discharged	37 (66%)	88 (73%)	
Died	1 (2%)	2 (2%)	

Fever was measured in degrees Celsius (°C). Data are median, *n* (range), or *n* (%). *P* values (from χ2 tests or Fisher exact tests) compared medical staff with no ICU hospitalized patients. *P* < 0.05 was considered statistically significant (in bold).

COVID-19, Coronavirus Disease 2019; CT, computed tomography; HCW, healthcare workers; ICU, intensive care unit; WRCH, Wuhan Red Cross Hospital.

## Increasing attention to healthcare workers

After the breakout of COVID-19 in Wuhan, the safety of medical staff has been a critical concern all over the world. For a long time, the medical personnel have made important contributions, promoting the development of world medical and health undertakings, working selflessly to improve the public health and protect people’s lives. During the process of controlling the current pandemic, the whole world is now committed to ensuring the prevention measures and working conditions of medical personnel and health institutions. Medical personnel is a typical close-contact group, so chest computerized tomography (CT) scans or virus RT-PCR screening should be employed to help identify and isolate potential SARS-CoV-2 infections. Otherwise, more medical staff would get cross-infected by their coworkers.

There were 4 main reasons for infections of medical personnel. First, human-to-human transmission of SARS-CoV-2 was not initially realized and people, including the medical staff, were not alerted of the severity of this new disease at the start of the pandemic. Second, hospital stocks of airborne precautions such as a fit-tested N95 respirator and other medical protective equipment were severely low and insufficient to deal with the outbreak. Third, many medical staff had to work more than 8 hours per day, with poor nutrition and insufficient sleep and under tremendous stress. The extreme working condition could have contributed to weakened immunity in the HCWs. Fourth, HCWs speculatively experienced more exposures, such as a higher frequency of interventional medical operations and aerosol-generating procedures. HCWs on the frontline of the pandemic are being pushed to their limits to provide patient care under enormous stress and PPE deficiency. Appropriate PPE is very effective at preventing COVID-19 infection in HCWs, and adequate training about proper donning and doffing of PPE is important, especially considering the worldwide PPE shortage [7]. A senior registered nurse was at the entrance of each designated COVID-19 ward to monitor HCW donning and doffing of PPE 24/7.

We are aware of the limitations of this study. First, earlier hospitalization may have contributed to milder disease manifestations in the medical staff group. Second, there were differences in age and background medical conditions between the HCWs group and nonmedical group. More studies are needed to get a more comprehensive understanding of medical staff with COVID-19.

## Conclusions

Medical professionals are at high risk of exposure to SARS-CoV-2; however, among our study groups, medical professionals displayed less severe manifestations of the disease than the nonmedical populations. Our experience and analysis of HCWs as COVID-19 patients in WRCH may help refine preventive strategies and reduce the anxiety of frontline HCWs.

## Declarations

### Ethics statement

This study was approved by the Ethics Committee of the Wuhan Red Cross Hospital. Formal verbal consent was obtained from participants.

### Consent for publication

Not applicable.

The lead authors and manuscript’s guarantor affirm that the manuscript is an honest, accurate, and transparent account of the study being reported; that no important aspects of the study have been omitted; and that any discrepancies from the study as planned have been explained.
